# Low status, humiliation, dopamine and risk of schizophrenia

**DOI:** 10.1017/S0033291722003816

**Published:** 2023-02

**Authors:** Jean Paul Selten, Johan Ormel

**Affiliations:** 1 University of Maastricht, School for Mental Health and Neuroscience, Maastricht, The Netherlands; 2 Rivierduinen Institute for Mental Health Care, Leiden, The Netherlands; 3 Department of Psychiatry, University of Groningen, University Medical Centre Groningen, Groningen, The Netherlands

## Abstract

The social defeat hypothesis of schizophrenia, which proposes that the chronic experience of outsider status or subordinate position leads to increased striatal dopamine activity and thereby to increased risk, has been criticized. The aims of this paper are to improve the definition of defeat and to integrate the social defeat hypothesis with the neurodevelopmental hypothesis. Marmot advanced the idea that low status is pathogenic in that it is associated with a lack of social participation and a lack of autonomy. Given the similarity with outsider status and subordinate position, we re-define social defeat as low status. From this new perspective it is also likely that pre-schizophrenic impairments (of neurodevelopmental origin or not) are pathogenic in that they contribute to low status. The effect of low status may be enhanced by repeated exposure to humiliation, but few studies have measured this variable. Since most individuals exposed to low status do not develop schizophrenia, we propose that this risk factor increases the risk of disorder in the presence of a poor homeostatic control of dopamine neurons in midbrain and dorsal striatum. This is consistent with studies of healthy subjects which report a negative association between low socio-economic status and dopamine D2/D3 receptor availability in the dorsal striatum. In this new version of the social defeat hypothesis we propose that the combination of low status, repeated humiliation and poor homeostatic control of dopamine neurons in midbrain and dorsal striatum leads to increased striatal dopamine activity and thereby to an increased risk of schizophrenia.

## Introduction

The social defeat hypothesis of schizophrenia proposes a common denominator for several risk factors (e.g. disadvantaged ethnic minority status, low IQ, childhood trauma, drug abuse, hearing impairment) and posits that the long-term experience of defeat, defined as an unwanted outsider status or a subordinate position, is one of the mechanisms that may lead to increased dopamine activity in the striatum and thereby to an increased risk (Selten & Cantor-Graae, 2005; Selten, van der Ven, Rutten, & Cantor-Graae, 2013). Further work demonstrated increased risks of psychosis for African-Americans, Australian Aboriginals, Maori in New Zealand (Bresnahan *et al*., 2007; Mirza *et al*., 2022; Petrovic-van der Deen *et al*., 2020) and for subjects with a non-heterosexual orientation, gender identity or autism spectrum disorder (e.g. Gevonden *et al*., 2014a; Hanna *et al*., 2019; Post, Veling & Group Investigators, 2021; Selten, Lundberg, Rai & Magnusson, 2014). The evidence of dopamine dysregulation in non-psychotic individuals exposed to defeating experiences, however, is mixed (e.g. Egerton *et al*., 2017; Schalbroeck *et al*., 2021).

The hypothesis has been criticized. Dykxhoorn and Kirkbride (2018) wrote: ‘While social defeat is an attractive hypothesis, it is yet to be operationalized in empirical research’. Schalbroeck (2020) criticized the hypothesis for an unclear definition of social defeat and for difficulties with its measurement. Fletcher and Birk (2021, 2022) consider the sociology in the social defeat hypothesis as ‘thin’ and observe that the psychological interface that bridges the social and the biological is underdeveloped.

The concept of social defeat has been derived from animal research. In our response to the above criticisms we will introduce the concepts of low status and humiliation. Is it possible to re-formulate the social defeat hypothesis using these concepts? Is it possible to define and measure them?

Another major issue concerns the relationship with influential versions of the neurodevelopmental hypothesis of schizophrenia, which posit that the cognitive, social and motor problems among pre-schizophrenic subjects are markers of a disturbance in the development of the brain (e.g. Howes & Murray, 2014; Jones, Rodgers, Murray & Marmot, 1994). Since the causes of this disturbance are widely considered to be mainly genetic (e.g. Hall & Bray, 2022), one could interpret social defeat or low status as just a ‘by-product’ of this disturbance. Is the social defeat hypothesis compatible with the neurodevelopmental hypothesis?

In addition, there is a phenotypic continuum and a strong genetic correlation (0.68; Brainstorm Consortium *et al*., 2018) between schizophrenia and bipolar disorder. Can the findings concerning low status inform us about differences in aetiology between the two disorders?

Finally, since most unsuccessful individuals do not develop schizophrenia, the social defeat hypothesis postulated that defeat increases risk when it leads to ‘an increased baseline activity of the mesolimbic dopamine system or to sensitization of this system’ (Selten & Cantor-Graae, 2005). Is this formulation up-to-date?

Here we respond to these issues and introduce a new version of the social defeat hypothesis.

## Low status and humiliation

Status has been defined as the respect, admiration and deference an individual is voluntarily afforded by others, based on that individual's perceived instrumental value (Anderson, Hildreth, & Howland, 2015). Important determinants of status are physical appearance, athleticism, intelligence, level of education, occupation, income, marital status and parenthood (Anderson *et al*., 2015; Vannatta, Gartstein, Zeller & Noll, 2009). Thus, status is not the same as socio-economic status (SES). The four times increased risks of schizophrenia for migrants from Africa or the Caribbean to Europe illustrate the importance of making this difference, because many of them experience a rise in income and a decline in status (Selten, van der Ven, Termorshuizen, 2020). Regrettably, most studies concern SES, which is easy to measure (level of education, income, occupation).

The desire for status is widely considered a fundamental human motive and a lack of status is a major risk factor for a large variety of diseases (Anderson *et al*., 2015; Marmot, 2005). The importance of status has also been recognized by the authors of the social production function theory, who posit that people ‘produce’ their social well-being by generating affection, status and behavioural confirmation (Ormel, Lindenberg, Steverink, & Vonkorff, 1997). In order to optimize this ‘production’, people choose and substitute their goals: if the resources for status achievement decrease, a person may, for instance, try to increase the ‘production’ of affection. Behavioural confirmation is approval for doing the right things. Intriguingly, the negative effect of low status on health is only in part explained by the usual risk factors for disease, such as smoking, hypertension, obesity and lack of exercise (e.g. Mackenbach *et al*., 2008; Marmot *et al*., 1991). Marmot (2005, 2005b) advanced the idea that low status is pathogenic in that it is associated with a lack of social participation and a lack of autonomy. The similarity with the pathogenic factors proposed by the social defeat hypothesis, viz. an unwanted outsider status or a subordinate position, supports our proposal to use low status in a new definition of defeat.

There are several reasons to consider *repeated humiliation* as a second factor of importance. Firstly, the pattern of the epidemiological findings shows the highest risks for the most rejected groups: individuals with black skin colour in Europe, subjects with gender dysphoria or low IQ (Becerra-Culqui *et al*., 2018; Dragon, Guerino, Ewald, & Laffan, 2017 Hanna *et al*., 2019; Khandaker, Barnett, White, & Jones, 2011; Selten *et al*., 2020). Secondly, one reason why a lack of social participation or autonomy is experienced as so stressful is the concomitant threat to one's self-esteem. Thirdly, individuals with an outsider status or subordinate position are beloved targets of aggression, because they are not in a position to respond. Finally, there is some evidence from studies of clinical and non-clinical samples. A randomized experiment in a non-clinical sample showed that exposure to negative feed-back and social exclusion led to paranoid ideation, a process mediated by low self-esteem (e.g. Kesting *et al*., 2013). An Experience Sampling and Monitoring (ESM)-study of a mixed clinical/nonclinical sample showed that a decrease in self-esteem was associated with an immediate increase in paranoia (Thewissen, Bentall, Lecomte, van Os, & Myin-Germeys, 2008). A recent study investigated three types of subjects (high level schizotypy, at risk mental state, first psychotic episode) and reported that low self-esteem, anxiety and sadness mediated the pathway from stress to psychotic-like experiences and paranoia in daily life (Monsonet, Rockwood, Kwapil, & Barrantes-Vidal, 2022).

However, the evidence of a role for humiliation is limited due to a lack of studies that measured it. Humiliation is difficult to measure, because many people deny the occurrence of degrading events or do not wish to report them. Some time ago researchers suggested that these difficulties might be addressed by the development of measures designed to uncover implicit self-esteem, but this project was not successful (Buhrmester, Blanton & Swann, 2011). A possibly valid instrument is the Life Events and Difficulties Schedule, which does not only collect information on the occurrence of life events, but also on their contextual meaning. The researchers reported that events in all likelihood followed by feelings of humiliation are associated with a far greater risk of depression than similar events without this context (Brown, Harris, & Hepworth, 1995). To the best of our knowledge large-scale research on the context of life events that occurred before the start of the prodromal period of schizophrenia has not yet been conducted. Another approach to examine any impact of humiliation is the above-mentioned ESM-method. It is possible to add variables that capture humiliating experiences, such as discrimination.

In the absence of widely accepted instruments for the measurement of humiliation, the assessment of social defeat is equivalent to the measurement of status. This can be done using peer-ratings, self-assessments, ranks within existing hierarchies and information on SES. One can collect peer ratings by inviting members of a relatively small group (e.g. students on a dorm) to rate each fellow group member on whether he or she is respected and admired. These studies show that individuals are highly accurate perceivers of others' status (reviewed by Anderson *et al*., 2015).

Although self-assessments are prone to self-serving biases, they appear to predict health status rather well. Several studies examined the value of subjective social status as a predictor of important health outcomes (e.g. angina, respiratory illness, diabetes, depression; scores on General Health Questionnaire) and the results suggested that subjective social status is often a better predictor of health than SES (Seeman, Stein Merkin, Karlamangla, Koretz & Seeman, 2014; Singh-Manoux, Adler & Marmot, 2003), perhaps due to a more precise and valid score of one's place in the social hierarchy (Singh-Manoux, Marmot, & Adler, 2005). The correlation between self-assessments of social status and peer-ratings is on average about *r* = 0.50 (Anderson *et al*., 2015). Of note, the outcome of a self-assessment depends on the choice of the referent group, which can be, for instance, other persons in society, neighbours or others of the same ethnic group (Wolff, Acevedo-Garcia, Subramanian, Weber & Kawachi, 2009). Although SES only weakly predicts subjective well-being and health, it is a useful proxy for status. In sum, the measurement of status is not easy, but feasible.

## Neurodevelopmental hypothesis

Many previous studies of pre-schizophrenic subjects attributed their low SES to the disease process and went on to discuss the SES of their parents (reviewed by Kwok, 2014). We think that the situation is more complex.

Indeed, a considerable body of evidence indicates that individuals who go on to develop schizophrenia score below average on social and motor functioning, intelligence and academic achievement before age 16 (e.g. Burton *et al*., 2016; Dickson, Laurens, Cullen & Hodgins, 2012; Dickson *et al*., 2020; Matheson *et al*., 2013). A well-known study, for instance, showed that they reach certain developmental milestones, like walking, significantly later than other children (Jones *et al*., 1994). While it should be noted that many patients do not exhibit such developmental abnormalities and that the predictive value of each developmental marker is low (Parellada, Gomez-Vallejo, Burdeus & Arango, 2017), many have already a diagnosis of a mental disorder at an early age. A prospective study of the Dunedin cohort reported that 52.8% of subjects diagnosed with schizophreniform disorder at age 26 had already been diagnosed with a mental disorder before age 15 (Kim-Cohen *et al*., 2003; see also Gyllenberg *et al*., 2010; 2003; Maibing *et al*., 2015). Given the negative consequences (e.g. problems in relationships, lower educational attainment, stigma), this probably means that many subjects who go on to develop schizophrenia have already a relatively low social status before age 16 (Kaushik, Kostaki & Kyriakopoulos, 2016; Kessler, Foster, Saunders & Stang, 1995).

In response to the question as to whether low status is just a by-product of a disturbance in neurodevelopment, we contend that a neurodevelopmental origin of these problems does not rule out the possibility that important consequences, viz. low status and humiliation, *become pathogenic factors in themselves*. It is highly unlikely that migrants from developing countries in Europe, African-Americans, Aboriginals in Australia, Maori in New Zealand, victims of bullying, people with a childhood trauma, hearing impairment, homosexual orientation, gender dysphoria or low IQ share a disorder in the development of their brain which explains their increased risk of schizophrenia. A causal contribution of low status and repeated humiliation is more likely.

Khandaker *et al*. (2011) argue that findings of premorbid IQ deficits constitute evidence of a neurodevelopmental contribution to the disorder, but it would be premature to conclude that this is the sole explanation for the association between IQ and risk. An additional explanation implies that low IQ may lead to low status and exposure to humiliation. This is important because genome-wide analyses show an extensive genetic overlap between schizophrenia and intelligence (Smeland *et al*. 2020).

## Bipolar disorder

Although a review concluded that impairments in neurodevelopment may play a role in a subset of individuals with bipolar disorder (i.e. those with an early age at onset and those exhibiting psychotic features), these impairments are generally less serious than in schizophrenia (Demjaha, MacCabe, & Murray, 2012; Kloiber *et al*., 2020). As for the main risk factors that inspired the social defeat hypothesis of schizophrenia (disadvantaged ethnic minority status, urban upbringing, low intelligence, childhood trauma), childhood trauma is the single one with a large impact on the risk for bipolar disorder (Zhang *et al*., 2020). Urban upbringing and low intelligence are not associated with an increased risk for bipolar disorder, while ethnic minority status is only weakly associated with this risk (Swinnen & Selten, 2007). Thus, the two disorders do not only differ with respect to neurodevelopment, but also with reference to pre-morbid social status: low in schizophrenia and average or above average in bipolar disorder. Consequently, it would be interesting to conduct a longitudinal study and to examine the unique contributions of status and neurodevelopmental impairment to the nature of the disorder that follows.

## Dopamine and status

The dopamine hypothesis is still the most important idea about the pathogenesis of schizophrenia (McCutcheon, Krystal & Howes, 2020; Weinstein, Chohan, Slifstein, Kegeles, Moore & Abi-Dargham, 2017). According to recent evidence the disorder is associated with increased synthesis and release of dopamine in the dorsal striatum and with hypo-activity in the cortex. Hyperdopaminergic states are widely believed to lead to aberrant assignment of salience and thereby to psychosis, but it should also be noted that striatal hyperactivity cannot be demonstrated in one third of patients (Brugger *et al*., 2020).

Before discussing any relationship between dopamine and status, we would like to point out that the results of studies on healthy monozygotic and dizygotic twins indicate a role for both genetic and environmental factors in the materialization of dopamine function in the striatum. An investigation of striatal dopamine synthesis capacity reported two values for heritability, depending on the type of analysis chosen, 0.44 and 0.33 (Stokes *et al*., 2013). A second study observed a heritability of 0.67 for the availability of striatal D2/D3 receptors in the striatum (Borg *et al*., 2016). Other studies of non-human primates and humans do report a relationship between status and dopamine function. A well-known study of individually and socially housed *cynomolgus macaques* found no difference in the amount or availability of dopamine D2/3 receptors during individual housing, while subsequent social housing increased this measure in the dominant monkeys and produced no change in the subordinate monkeys (Morgan *et al*. 2002). Importantly, when the social ranks were manipulated by placing them into new social groups, previously subordinate monkeys showed significant increases in D2/3 receptor availability in the striatum, with the largest increase observed in those that became dominant after reorganization (Czoty, Gould, Gage, & Nader, 2017).

As for humans, a study of healthy volunteers reported a positive correlation between SES and dopamine D2/D3 receptor availability in the striatum (*r* = 0.71; *p* = 0.004) and a similar association between social support and this availability (*r* = 0.73; *p* = 0.02) (Martinez *et al*., 2010). (A greater dopamine D2/D3 receptor availability can be interpreted as an increase in these receptors or in a decrease of endogenous dopamine release.) A larger study reported weaker correlations, but extended the finding by showing a correlation between SES and the availability of dopamine D2/D3 receptors in the *dorsal* striatum (caudate *r* = .35; *p* = 0.024; putamen *r* = 0.39; *p* = 0.11), not in the ventral striatum (*p* = 0.61) (Wiers *et al*., 2016). Of note, this New York group also reported a negative association between genetic markers of African ancestry and dopamine receptor availability in the dorsal striatum (Wiers *et al*., 2018), while the association between markers of European ancestry and this availability was positive. The authors note that these findings could be due to racial differences or differences in social status. We conclude that there is evidence of an association between low SES and increased dopaminergic activity in the dorsal striatum of humans.

## Dopamine dysregulation

How to explain the fact that most individuals who experience low status and repeated humiliation do *not* develop schizophrenia? The simplest explanation is that their midbrain or striatal dopamine neurons do not develop an increased activity in response to these stressors. While many factors contribute to the homoeostatic control of these neurons, the precise mechanisms are incompletely understood. As for the dopamine system, research findings have not implicated genes directly involved in determining dopamine synthesis and release (McCutcheon *et al*., 2020; Weinstein *et al*., 2017). The activity of dopamine neurons in midbrain and striatum appears to be controlled by several factors, including inhibitory glutamatergic projections, inhibitory parvalbumin-(PV)-expressing interneurons, the hypothalamic-pituitary-adrenal axis and drug abuse (Howes, McCutcheon, Owen & Murray, 2017; Kelly & Fudge, 2018; Murray *et al*., 2017). Some evidence suggests that these mechanisms are impaired in schizophrenia: the risk of disorder is associated with genes involved in the development and maintenance of glutamatergic synapses (Schizophrenia Working Group of the Psychiatric Genomics Consortium, 2014) and schizophrenia is associated with a reduction of interneurons in prefrontal and cingulate cortices (Benes, McSparren, Bird, SanGiovanni & Vincent, 1991). Grace (2016) suggested, therefore, that impaired function of PV-expressing interneurons in cortex or hippocampus may lead to disinhibition of meso-striatal dopamine neuron activity via a polysynaptic pathway.

The results of two animal studies suggest a contribution of epigenetic factors. Krishnan *et al*. (2007) demonstrated that an inbred population of mice subjected to social defeat can be separated into susceptible and unsusceptible populations that differ along several physiological domains. Differences in ventral tegmental area dopamine neuron firing rates, for instance, were associated with vulnerability and insusceptibility. Since there was little genetic variation between the mice, the authors conclude that epigenetic factors are important. The authors of the second study devised a large environment (‘Souris City’), where inbred mice lived continuously in large groups, and showed that the divergence in individual behaviours was mirrored by developing differences in midbrain dopamine neuron firing properties, which were, again, not explained by genetic diversity (Torquet *et al*., 2018).

In sum, there are good reasons to hypothesize that some individuals suffer from an inability to control the activity of dopamine neurons in midbrain and dorsal striatum and develop dopamine sensitization, whereby the experience of low status (and perhaps humiliation) contributes to the progressive amplification of a dopamine response.

## A new version of the social defeat hypothesis

In a new version of the social defeat hypothesis we propose that the combination of low status, repeated humiliation and a low homoeostatic control of dopamine neurons in midbrain and dorsal striatum leads to increased striatal dopamine activity and thereby to an increased risk of schizophrenia. The biochemistry of the disorder is, of course, a lot more complicated. Nonetheless, we think it is important to propose a bridge between the psychology and the biology of the disorder and to test the validity of this bridge. The results of investigations will lead to more refined hypotheses. As for an inability to acquire status one can distinguish between a lack of talents or endowments and exposure to discrimination or high levels of socio-economic inequality.

One can distinguish several differences with another important hypothesis in this area, which proposes a socio-developmental pathway to psychosis (e.g. Morgan, Charalambides, Hutchinson & Murray, 2010; Morgan, Knowles, & Hutchinson, 2019). The authors emphasize exposure to social adversity and trauma (rather than low status and humiliation) and propose that this exposure ‘impacts on neurobiological development (in particular the stress response and dopamine systems) to create an enduring liability to psychosis’ (Morgan *et al*., 2019). Thus, this hypothesis places more emphasis on external factors (adversity) and does not clearly specify a biological mechanism. Moreover, while low status plus repeated humiliation is definitely a form of adversity, there are many types of adversity. Thus, the social defeat hypothesis refers to a more circumscribed area of experience.

One could criticize the hypothesis for being unspecific, as low status (or: low SES) is also a risk factor for many other psychiatric disorders, like depression and addiction (e.g. Swendsen *et al*., 2009; Taylor, Gooding, Wood & Tarrier, 2011). However, low status is, like cigarette smoking, an important risk factor for disease in general. Bipolar disorder is probably an exception to this rule, while schizophrenia is not (Coryell, Endicott, Keller, Andreasen, Grove, Hirschfeld *et al*., 1989; Goodwin & Jamison, 2007).

## Testing the hypothesis

First, in order to test the idea that low status is a risk factor, it is important to measure it. Second, it is important to develop better methods to measure the experience of humiliation. If this turns out to be impossible, it is preferable to remove this variable from the hypothesis. Third, longitudinal studies are required to examine whether status, or a change in status have an impact on dopamine function. One could, for instance, follow recently arrived migrants from developing countries. Fourth, more high-quality investigations of dopamine function in non-psychotic individuals exposed to humiliating experiences are needed. It is important to understand why the results of these studies are mixed (e.g. Bloomfield, McCutcheon, Kempton, Freeman & Howes, 2019; Egerton *et al*., 2016, 2017; Gevonden *et al*., 2014b; Schalbroeck *et al*., 2021). Finally, we would like to observe that elucidation of the mechanisms whereby low status causes disease in general might increase our insight into the pathogenesis of schizophrenia.

## Supplementary material

For supplementary material accompanying this paper visit https://doi.org/10.1017/S0033291722003816. click here to view supplementary material
