# Supplementary material for: Low status, humiliation, dopamine and risk of schizophrenia
Source: Psychol Med. 2023 Jan 25;53(3):609–13. doi: 10.1017/S0033291722003816 (PMC9976000; doi:10.1017/S0033291722003816)
Supplement: Supplementary file 1 [file S0033291722003816sup001.docx]

**Supplementary References PSM-D-22-01294**

Becerra-Culqui, T.A., Liu, Y., Nash, R., Cromwell, L., Flanders, W.D., Getahun, D., …, Goodman M. (2018). Mental Health of Transgender and Gender Nonconforming Youth Compared With Their Peers. *Pediatrics*, *141*(5):e20173845. doi: 10.1542/peds.2017-3845.

Benes, F.M., McSparren, J., Bird, E.D., SanGiovanni, J.P., & Vincent, S.L. (1991). Deficits in small interneurons in prefrontal and cingulate cortices of schizophrenic and schizoaffective patients. *Archives of General Psychiatry*, *48*(11), 996-1001. doi: 10.1001/archpsyc.1991.01810350036005.

Bloomfield, M.A., McCutcheon, R.A., Kempton, M., Freeman, T.P. & Howes, O. (2019). The effects of psychosocial stress on dopaminergic function and the acute stress response. *Elife,* Nov 12;8:e46797. doi: 10.7554/eLife.46797.

Borg, J., Cervenka, S., Kuja-Halkola, R., Matheson, G.J., Jönsson, E.G., Lichtenstein, P., … Farde, L. (2016). Contribution of non-genetic factors to dopamine and serotonin receptor availability in the adult human brain. *Molecular Psychiatry, 21*(8), 1077-84. doi: 10.1038/mp.2015.147.

Brainstorm Consortium, Anttila, V., Bulik-Sullivan, B., Finucane, H.K., Walters, R.K., Bras, J., …Murray, R. (2018). Analysis of shared heritability in common disorders of the brain. *Science, 360*(6395), eaap8757. doi: 10.1126/science.aap8757.

Bresnahan, M., Begg, M.D., Brown, A., Schaefer, C., Sohler, N., Insel, B., … Susser, E. (2007). Race and risk of schizophrenia in a US birth cohort: another example of health disparity? *International Journal of Epidemiology, 36*(4), 751-8. doi: 10.1093/ije/dym041.

Brugger, S.P., Angelescu, I., Abi-Dargham, A., Mizrahi, R., Shahrezaei, V. & Howes, O.D. (2020). Heterogeneity of Striatal Dopamine Function in Schizophrenia: Meta-analysis of Variance. *Biological Psychiatry, 87*(3), 215-224. doi: 10.1016/j.biopsych.2019.07.008. Erratum in: *Biological Psychiatry,* 87(3), 305.

Buhrmester, M.D., Blanton, H., Swann, & W.B. Jr. (2011). Implicit self-esteem: nature, measurement, and a new way forward. *Journal of Personality and Social Psychology, 100*(2), 365-85. doi: 10.1037/a0021341.

Burton, B.K., Hjorthøj, C., Jepsen, J.R., Thorup, A., Nordentoft, M., & Plessen, K.J. (2016). Research Review: Do motor deficits during development represent an endophenotype for schizophrenia? A meta-analysis. *Journal of Child Psychology and Psychiatry,* *57*(4), 446-56.

Coryell, W., Endicott, J., Keller, M., Andreasen, N., Grove, W., Hirschfeld, R.M., & Scheftner, W. (1989). Bipolar affective disorder and high achievement: a familial association. *American Journal of Psychiatry, 146(8),* 983-8. doi: 10.1176/ajp.146.8.983.

Dickson, H., Laurens, K.R., Cullen, A.E. & Hodgins, S. (2012). Meta-analyses of cognitive and motor function in youth aged 16 years and younger who subsequently develop schizophrenia. *Psychological Medicine,* *42*(4), 743-55. doi: 10.1017/S0033291711001693.

Dickson, H., Hedges, E.P., Ma, S.Y., Cullen, A.E., MacCabe, J.H., Kempton, M.J., …Laurens, K.R. (2020). Academic achievement and schizophrenia: a systematic meta-analysis*. Psychological Medicine, 50*(12), 1949-1965. doi: 10.1017/S0033291720002354.

Dykxhoorn, J. & Kirkbride, J.B. (2018). Psychoses sans Frontieres: towards an interdisciplinary understanding of psychosis risk amongst migrants and their descendants. *Epidemiology and Psychiatric Sciences, 28*(2), 146-152. doi: 10.1017/S2045796018000501.

Egerton, A., Valmaggia, L.R., Howes, O.D., Day, F., Chaddock, C.A., Allen, P., … McGuire, P. (2016). Adversity in childhood linked to elevated striatal dopamine function in adulthood. *Schizophrenia Research*, *176*(2-3), 171-176. doi: 10.1016/j.schres.2016.06.005.

Egerton, A., Howes, O.D., Houle, S., McKenzie, K., Valmaggia, L.R., Bagby, M.R., ….., Mizrahi, R. (2017). Elevated Striatal Dopamine Function in Immigrants and Their Children: A Risk Mechanism for Psychosis. *Schizophrenia Bulletin*, *43*(2), 293-301. doi: 10.1093/schbul/sbw181.

Fletcher, J.R. & Birk, R.H. (2021). From fighting animals to the biosocial mechanisms of the human mind: a comparison of Selten’s social defeat and Mead’s symbolic interaction. *Sociological Review, 68*(6), 1273-1289.

Fletcher, J.R. & Birk, R.H. (2022). The conundrum of the psychological interface: on the problems of bridging the biological and the social. *History of Human Sciences*, *35*(3-4), 317-39.

Gevonden, M.J., Selten, J.P., Myin-Germeys, I., de Graaf, R., ten Have, M., van Dorsselaer, S, … Veling, W. (2014a). Sexual minority status and psychotic symptoms: findings from the Netherlands Mental Health Survey and Incidence Studies (NEMESIS). *Psychological Medicine, 44*(2), 421-33. doi: 10.1017/S0033291713000718.

Gevonden, M., Booij, J., van den Brink, W., Heijtel, D., van Os, J. & Selten, J.P. (2014b). Increased release of dopamine in the striata of young adults with hearing impairment and its relevance for the social defeat hypothesis of schizophrenia. *JAMA Psychiatry, 71*(12), 1364-72. doi: 10.1001/jamapsychiatry.2014.1325.

Goodwin, F.K., Jamison, K.R. (2007). *Manic-depressive illness*. New York: Oxford University Press, pp. 181-182.

Grace, A.A. (2016). Dysregulation of the dopamine system in the pathophysiology of schizophrenia and depression. *Nature Reviews Neuroscience,* *17*(8), 524-32. doi: 10.1038/nrn.2016.57.

Gyllenberg, D., Sourander, A., Niemelä, S., Helenius, H., Sillanmäki, L., Piha, J., … Almqvist, F (2010). Childhood predictors of later psychiatric hospital treatment: findings from the Finnish 1981 birth cohort study. *European Child and Adolescent Psychiatry, 19*(11), 823-33. doi: 10.1007/s00787-010-0129-1.

Hall, J. & Bray, N.J. (2022). Schizophrenia Genomics: Convergence on Synaptic Development, Adult Synaptic Plasticity, or Both? *Biological Psychiatry, 91*(8), 709-717. doi: 10.1016/j.biopsych.2021.10.018.

Hanna, B., Desai, R., Parekh, T., Guirguis, E., Kumar, G. & Sachdeva, R. (2019). Psychiatric disorders in the U.S. transgender population (20. *Annals of Epidemiology*, *39*, 1-7.e1. doi: 10.1016/j.annepidem.2019.09.009.

Howes, O.D. & Murray, R.M. (2014). Schizophrenia: an integrated sociodevelopmental-cognitive model. *Lancet,* *383*(9929), 1677-1687. doi: 10.1016/S0140-6736(13)62036-X.

Howes, O.D., McCutcheon, R., Owen, M.J. & Murray, R.M. (2017). The Role of Genes, Stress, and Dopamine in the Development of Schizophrenia. *Biological Psychiatry 81*(1), 9-20. doi: 10.1016/j.biopsych.2016.07.014.

Jones, P., Rodgers, B., Murray, R. & Marmot, M. Child development risk factors for adult schizophrenia in the British 1946 birth cohort. *Lancet, 344*(8934), 1398-402. doi: 10.1016/s0140-6736(94)90569-x.

Kaushik, A., Kostaki, E., & Kyriakopoulos M. (2016). The stigma of mental illness in children and adolescents: A systematic review. *Psychiatry Research, 243,* 469-94. doi: 10.1016/j.psychres.2016.04.042. Epub 2016 Jun 23.

Kelly, E.A. & Fudge, J.L. (2018). The neuroanatomic complexity of the CRF and DA systems and their interface: What we still don't know. *Neuroscience and Biobehavioral Reviews*, *90*, 247-259. doi: 10.1016/j.neubiorev.2018.04.014.

Kessler, R.C., Foster, C.L., Saunders, W.B. & Stang, P.E. (1995). Social consequences of psychiatric disorders, I: Educational attainment. *American Journal of Psychiatry, 152(7),* 1026-32. doi:10.1176/ajp.152.7.1026.

Kesting, M.L., Bredenpohl, M., Klenke, J., Westermann, S., & Lincoln, T.M. (2013). The impact of social stress on self-esteem and paranoid ideation. *Journal of Behavior Therapy and Experimental Psychiatry, 44(1),* 122-8. doi: 10.1016/j.jbtep.2012.07.010. Epub 2012 Aug 11.

Krishnan, V., Han, M.H., Graham, D.L., Berton, O., Renthal, W., Russo, S.J., … Nestler, E.J. (2007). Molecular adaptations underlying susceptibility and resistance to social defeat in brain reward regions. *Cell, 131*(2), 391-404. doi: 10.1016/j.cell.2007.09.018.

Mackenbach, J.P., Stirbu, I., Roskam, A.J., Schaap, M.M., Menvielle, G., Leinsalu, M., … European Union Working Group on Socioeconomic Inequalities in Health (2008). Socioeconomic inequalities in health in 22 European countries. *New England Journal of Medicine* *358*(23), 2468-81. doi: 10.1056/NEJMsa0707519.

Maibing, C.F., Pedersen, C.B., Benros, M.E., Mortensen, P.B., Dalsgaard, S. & Nordentoft, M. (2015). Risk of Schizophrenia Increases After All Child and Adolescent Psychiatric Disorders: A Nationwide Study. *Schizophrenia Bulletin*, *41*(4), 963-70. doi: 10.1093/schbul/sbu119.

Marmot, M.G., Smith, G.D., Stansfeld, S., Patel, C., North, F., Head, J., … Feeney, A. (1991). Health inequalities among British civil servants: the Whitehall II study. *Lancet, 337*(8754), 1387-93. doi: 10.1016/0140-6736(91)93068-k.

Marmot, M.G. (2005b). Status syndrome: a challenge to medicine. *JAMA,* *295*(11), 1304-7. doi: 10.1001/jama.295.11.1304.

Matheson, S.L., Vijayan, H., Dickson, H., Shepherd, A.M., Carr, V.J., & Laurens, K.R. (2013). Systematic meta-analysis of childhood social withdrawal in schizophrenia, and comparison with data from at-risk children aged 9-14 years. *Journal of Psychiatric Research, 47*(8), 1061-8. doi: 10.1016/j.jpsychires.2013.03.013.

McCutcheon, R.A., Krystal, J.H., & Howes, O.D. (2020). Dopamine and glutamate in schizophrenia: biology, symptoms and treatment. *World Psychiatry, 19*(1), 15-33. doi: 10.1002/wps.20693. PMID: 31922684;

Mirza, T., Taft, W., He, V.Y., Gooding, J., Dingwall, K. & Nagel, T. (2022). Incidence of treated first-episode psychosis amongst Aboriginal and non-Aboriginal youth in the Top End of the Northern Territory, Australia. *Australasian Psychiatry*, *30*(4), 513-7. doi: 10.1177/10398562221075193. Epub ahead of print.

Morgan, C., Charalambides, M., Hutchinson, G. & Murray, R.M. (2010). Migration, ethnicity, and psychosis: toward a sociodevelopmental model. *Schizophrenia Bulletin*, *36*(4), 655-64. doi: 10.1093/schbul/sbq051.

Murray RM, Englund A, Abi-Dargham A, Lewis DA, Di Forti M, Davies C, … D'Souza DC (2017). Cannabis-associated psychosis: Neural substrate and clinical impact. *Neuropharmacology*, *124*, 89-104. doi: 10.1016/j.neuropharm.2017.06.018.

Parellada, M., Gomez-Vallejo, S., Burdeus, M. & Arango, C (2017). Developmental Differences Between Schizophrenia and Bipolar Disorder. *Schizophrenia Bulletin*, *43*(6):1176-1189. doi: 10.1093/schbul/sbx126.

Petrović-van der Deen, F.S., Cunningham, R., Manuel, J., Gibb, S., Porter, R.J., Pitama, S., … Lacey, C. (2020). Exploring indigenous ethnic inequities in first episode psychosis in New Zealand - A national cohort study. *Schizophrenia Research*, *223*, 311-318. doi: 10.1016/j.schres.2020.09.004.

Post, D., Veling W., & GROUP investigators (2021). Sexual minority status, social adversity and risk for psychotic disorders-results from the GROUP study. *Psychological Medicine, 51*(5), 770-776. doi: 10.1017/S0033291719003726.

Rothman, K. (2002). *Epidemiology. An introduction*. New York: Oxford University Press.

Schalbroeck, R. (2020). The social defeat hypothesis of schizophrenia: a parsimonious explanation for multiple psychosis risk factors? *Psychological Medicine,* doi: 10.1017/S0033291720004092. Epub ahead of print. PMID: 33168111.

Schalbroeck, R., van Velden, F.H.P., de Geus-Oei, L.F., Yaqub, M., van Amelsvoort, T., Booij, J. & Selten, J.P. (2021). Striatal dopamine synthesis capacity in autism spectrum disorder and its relation with social defeat: an [^18^F]-FDOPA PET/CT study. *Translational Psychiatry*, *11*(1), 47. doi: 10.1038/s41398-020-01174-w.

Schizophrenia Working Group of the Psychiatric Genomics Consortium. Biological insights from 108 schizophrenia-associated genetic loci (2014). *Nature, 511*(7510), 421-7. doi: 10.1038/nature13595.

Seeman, M., Stein Merkin, S., Karlamangla, A., Koretz, B. & Seeman, T. (2014). Social status and biological dysregulation: the "status syndrome" and allostatic load. *Social Science and Medicine, 118,* 143-51. doi: 10.1016/j.socscimed.2014.08.002.

Selten, J.P., Lundberg, M., Rai, D. & Magnusson, C. (2015). Risks for nonaffective psychotic disorder and bipolar disorder in young people with autism spectrum disorder: a population-based study. *JAMA Psychiatry, 72*(5), 483-9. doi: 10.1001/jamapsychiatry.2014.3059.

Selten, J.P., van der Ven, E. & Termorshuizen, F. (2020). Migration and psychosis: a meta-analysis of incidence studies. *Psychological Medicine*, *50*(2), 303-313. doi: 10.1017/S0033291719000035.

Singh-Manoux, A., Adler, N.E. & Marmot, M.G. (2003). Subjective social status: its determinants and its association with measures of ill-health in the Whitehall II study. *Social Science and Medicine, 56*(6), 1321-33. doi: 10.1016/s0277-9536(02)00131-4.

Stokes, P.R., Shotbolt, P., Mehta, M.A., Turkheimer, E., Benecke, A., Copeland, C., … Howes, O.D. (2013). Nature or nurture? Determining the heritability of human striatal dopamine function: an [18F]-DOPA PET study. *Neuropsychopharmacology*, *38*(3), 485-91. doi: 10.1038/npp.2012.207. PMID: 23093224;

Swinnen, S.G. & Selten, J.P. (2007). Mood disorders and migration: meta-analysis. *British Journal of Psychiatry, 190*, 6-10. doi: 10.1192/bjp.bp.105.020800.

Swendsen, J., Conway, K.P., Degenhardt, L., Dierker, L., Glantz, M., Jin, R. …. Kessler, R.C. (2009). Socio-demographic risk factors for alcohol and drug dependence: the 10-year follow-up of the national comorbidity survey. *Addiction 104(8),* 1346-55. doi: 10.1111/j.1360-0443.2009.02622.x. Epub 2009 Jun 22.

Taylor, P.J., Gooding, P., Wood, A.M. & Tarrier, N. (2011). The role of defeat and entrapment in depression, anxiety, and suicide. *Psychological Bulletin, 137*(3), 391-420. doi: 10.1037/a0022935.

Torquet, N., Marti, F., Campart, C., Tolu, S., Nguyen, C., Oberto, V., … Faure, P. (2018). Social interactions impact on the dopaminergic system and drive individuality. *Nature Communications*, *9*(1), 3081. doi: 10.1038/s41467-018-05526-5.

Vannatta, K., Gartstein, M.A., Zeller, M. & Noll, R.B. (2009). Peer acceptance and social behaviour during childhood and adolescence: how important are appearance, athleticism, and academic competence? *International Journal of Behavioral Development, 33*, 303-311.

Weinstein, J.J., Chohan, M.O., Slifstein, M., Kegeles, L.S., Moore, H., & Abi-Dargham, A. (2017). Pathway-Specific Dopamine Abnormalities in Schizophrenia. *Biological Psychiatry*, *81*(1), 31-42. doi: 10.1016/j.biopsych.2016.03.2104.

Wolff, L.S., Acevedo-Garcia, D., Subramanian, S.V., Weber, D. & Kawachi, I. (2009). Subjective Social Status, a New Measure in Health Disparities Research: Do Race/Ethnicity and Choice of Referent Group Matter? *Journal of Health Psychology*, *15*(4), 560-574..doi.org/10.1177%2F1359105309354345

Zhang, S., Lin, X., Yang, T., Zhang, S., Pan, Y., Lu, J. & Liu, J. (2020). Prevalence of childhood trauma among adults with affective disorder using the Childhood Trauma Questionnaire: A meta-analysis. *Journal of Affective Disorders*, *276*, 546-554. doi: 10.1016/j.jad.2020.07.001.
